# Procalcitonin for the diagnosis of invasive candidiasis: what is the evidence?

**DOI:** 10.1186/s40560-017-0252-x

**Published:** 2017-09-25

**Authors:** Santi Maurizio Raineri, Andrea Cortegiani, Filippo Vitale, Pasquale Iozzo, Antonino Giarratano

**Affiliations:** Department of Biopathology and Medical Biotechnologies (DIBIMED). Section of Anesthesia, Analgesia, Intensive Care and Emergency. Policlinico P. Giaccone, University of Palermo, Via del vespro 129, 90127 Palermo, Italy

**Keywords:** Invasive candidiasis, *Candida* spp., Procalcitonin, Sepsis

## Abstract

Procalcitonin is a widely used marker for the evaluation of infection and sepsis and to guide antibiotic therapy. During the last decade, several studies evaluated its role and diagnostic performance as a surrogate marker for the identification of *Candida* spp. in suspected invasive candidiasis. A low serum level and a favorable negative predictive value are the main findings for procalcitonin in this setting. The aim of this report is to provide an updated brief summary of the evidence supporting the use of PCT for the management of invasive candidiasis.

We read with interest the comprehensive review by Vijayan et al. about the role of procalcitonin (PCT) as a marker of sepsis and guide for antibiotic therapy [1]. However, we believe that another important role of PCT should be emphasized in light of recent literature, namely its usefulness in suspected invasive candidiasis (IC).

IC is a frequent cause of infection and sepsis in critically ill patients and is characterized by high morbidity, mortality, and costs [2–4]. The outcome of patients with IC seems to be associated with timing of antifungal treatment initiation [2, 5]. However, the turnaround time to the microbiological diagnosis of IC is long, ranging from 3 to 7 days [3]. Therefore, clinicians should often prescribe antifungals before definitive microbiological isolation of *Candida* spp. [6]. In fact, untargeted antifungal treatment is frequently used in clinical practice with high associated costs and potential risk of resistance [6]. In this regard, desirable features of a marker might be the ability to help in confirming or excluding IC when suspected and to differentiate between bacterial and fungal infection/sepsis in order to optimize antimicrobial treatment with a favorable cost-benefit balance.

In 2006, Charles et al. published a retrospective study enrolling 50 nonsurgical septic patients with positive blood cultures, 35 with bacteremia and 15 with candidemia [7]. They found a significantly lower PCT level in patients with candidemia (median 0.65 ng/ml) compared to those with bacteremia (median 9.75 ng/ml). PCT level higher than 5.5 ng/ml demonstrated a 100% negative predictive value (NPV) and 65% positive predictive value (PPV) for sepsis caused by *Candida* spp. Martini et al. performed a prospective study enrolling 48 critically ill surgical patients with sepsis and risk of fungal infection [8]. PCT levels were lower in patients with candidemia (median 0.71 [IQR 0.5–1.1]) than in those with bacteremia (median 12.9 [IQR 2.6–81.2]). Recently, Cortegiani et al. performed a retrospective study evaluating 260 diagnostic episodes from 182 patients (60% surgical) [9]. The aim of this study was to assess the PCT level in septic patients with *Candida*, bacterial or mixed bloodstream infection (BSI) evaluated with blood culture (BC), and a polymerase chain reaction (PCR) test. A significantly lower level of PCT was found in *Candida* BSI (median 0.99 ng/ml [IQR 0.86–1.34]) than in BSI caused by bacteria (median 16.7 ng/ml [IQR 7.52–50.2]) or in mixed BSI (median 4.76 ng/ml [IQR 2.98–6.08]). Consensual results were found for PCR results. A cut-off of ≤ 6.08 ng/ml for PCT yielded a sensitivity of 86.8%, a specificity of 87.6%, a PPV of 63.9%, and a NPV of 96.3%. Interestingly, more subsequent episodes of BSI were due to *Candida* compared to the first ones. It is known that subsequent episode of nosocomial infections is often characterized by an impaired (hypoergic) immunity and *Candida* spp. infections are often involved [6]. Authors speculated that this might have been one of the reasons explaining the lower level of PCT in patients with *Candida* BSI, stressing a link between the low activation of the immunity, isolation of *Candida* in blood, and low PCT level [9]. Very recently, Pieralli et al. performed a retrospective case-control study enrolling 64 septic patients with candidemia and 128 septic patients with bacteremia admitted in 3 internal medicine units [10]. Again, PCT levels were lower in patients with candidemia (median 0.73 ng/ml [IQR 0.26–1.85]) compared to those in patients with bacteremia (median 4.48 ng/ml [IQR 1.10–18.26]). PCT higher than 2.5 ng/ml had a NPV of 98.3% with an area under the curve (AUC) of 0.76 (0.68–0.84%) for identification of *Candida* spp. in blood. Lastly, the diagnostic performance of PCT was evaluated in association to (1-3)-beta-d-glucan (BDG), one of the widely used surrogate markers for fungal infection. Giacobbe et al. performed a retrospective study enrolling 166 patients from 3 intensive care units (ICUs), 73 with candidemia and 93 with bacteremia (almost 30% surgical and 60% medical) [11]. Patients with candidemia had lower PCT value (median 0.76 vs 4.32 ng/ml) than those with bacteremia. Authors evaluated together the diagnostic performance of BDG and PCT using a commonly recognized cut-off value of BDG for *Candida* identification (≥ 80 pg/ml) and the rounded best cut-off calculated in their population for PCT in candidemia (2 ng/ml). When both markers indicated candidemia, they showed higher PPV (96%) compared to 79 and 66% for BDG and PCT. When both indicated bacteremia, their NPV was similar to that of BDG alone (95 vs 93%).

Although several studies demonstrated the correlation between a low PCT level (< 2 ng/ml) and *Candida* infection and high NPV of PCT for *Candida* isolation, its role in management of antifungal treatment is far from established mainly because of the limitations in study design of supporting literature. A recently published research agenda on invasive fungal infections reported the “Utilization of PCT to guide treatment initiation and duration” as one of the ten priority for future trials in the field [12]. Meanwhile, clinicians might evaluate a PCT level in an infected/septic patient with risk factors for invasive candidiasis as one of the element to assess the probability of subsequent *Candida* isolation.

## Abbreviations

AUC: Area under the curve
BC: Blood culture
BDG: (1-3)-beta-d-glucan
BSI: Bloodstream infection
IC: Invasive candidiasis
NPV: Negative predictive value
PCR: Polymerase chain reaction
PCT: Procalcitonin
PPV: Positive predictive value
